# A Porosity-Dependent Constitutive Model for Concrete Under Wetting–Drying Cycles: Experimental and Field Validation

**DOI:** 10.3390/ma19173686

**Published:** 2026-08-30

**Authors:** Xiaozhong Zhang, Guomin Sun, Tao Li, Jiajun Shu

**Affiliations:** 1 School of Civil and Architectural Engineering, Guizhou University of Engineering Science, Bijie 551700, China; 2 The Key Laboratory of Smart Monitoring for Bridges in the Mountainous Area of Bijie City, Bijie 551700, China; 3 School of Mechanics and Civil Engineering, China University of Mining and Technology-Beijing, Beijing 100083, China

**Keywords:** wetting–drying exposure, concrete, constitutive model, experimental validation, field validation

## Abstract

The degradation of concrete under wetting–drying cycles significantly compromises the safety and durability of bridge structures; however, existing constitutive models often exhibit limitations in practical engineering applicability. To improve the engineering applicability of existing constitutive models, this study proposes a porosity-dependent uniaxial compressive constitutive model for concrete located in the wetting–drying zones of in-service bridge piers. This model is theoretically grounded in the strain equivalence principle and the Weibull statistical distribution. To validate the theoretical framework, six groups of concrete specimens with varying target porosities (15%, 20%, and 25%) were subjected to 15 consecutive 30-day sulfate wetting–drying exposure intervals, corresponding to a total exposure duration of 450 days. The macroscopic evolutions of porosity, mass variation, permeability coefficients, and uniaxial compressive stress–strain behavior were systematically evaluated. Furthermore, an independent field validation was conducted utilizing core samples extracted from the Saiqi Bridge, an in-service structure exposed to natural water-level fluctuations over a 25-year service period. The experimental results indicate that the theoretical stress–strain relationships predicted by the proposed model are in good agreement with the empirical measurements. In the field application, the core data revealed a reduction in concrete compressive strength from the initial design value of 40 MPa to 38.2 MPa. The porosity inversely predicted by the proposed model (16.9%) showed good agreement with the actual measured porosity (17%) of the bridge piers. By explicitly incorporating pore characteristics, the proposed model characterizes the mechanical deterioration of concrete. Consequently, it provides theoretical support for the performance assessment, numerical simulation, and structural strengthening of in-service bridges exposed to repeated wetting–drying exposure.

## 1. Introduction

Wetting-drying cycles constitute one of the primary degradation mechanisms affecting concrete in natural service environments. Driven by physicochemical actions—such as pore water pressure fluctuations and swelling induced by salt crystallization—microcracks within the concrete continuously initiate, propagate, and coalesce. This cumulative damage results in the progressive deterioration of the material’s mechanical properties, thereby directly compromising both structural safety and long-term durability [1]. Consequently, establishing a constitutive model capable of accurately characterizing the mechanical behavior of concrete subjected to wetting–drying cycles holds significant theoretical value and practical engineering relevance. Such a model is important for the reliable design, performance evaluation, and structural rehabilitation of hydraulic, harbor, and bridge infrastructure frequently exposed to fluctuating aqueous environments [2,3].

Currently, researchers have conducted multi-scale studies from both macroscopic and mesoscopic perspectives, leading to the development of three primary methodological approaches: empirical fitting models, damage mechanics models, and multi-field coupling models [4]. The trajectory of this research is increasingly shifting toward practical engineering applications and enhanced model refinement. Macroscopic investigations demonstrate that as the number of wetting–drying cycles increases, the peak strength and elastic modulus of concrete progressively decline, the initial compaction stage of the stress–strain curve is extended, and both plastic and residual deformations accumulate [5,6]. Driven by these observed behaviors, scholars have modified standard theoretical frameworks by incorporating phenomenological parameters, such as cycle count and moisture content, to formulate various empirical constitutive relationships [3]. While these empirical models are mathematically straightforward and convenient to implement, their practical applicability is often constrained by specific experimental conditions, limiting their broader applicability in diverse engineering scenarios.

Damage mechanics currently provides the mainstream theoretical framework for constitutive modeling. Within the context of continuum damage mechanics, material degradation induced by wetting–drying cycles is mathematically characterized through the use of damage variables [7]. By integrating statistical distributions with plastic flow criteria, researchers have developed statistical damage and plastic damage models. These models can be readily embedded into finite element programs to simulate damage evolution and describe the coupled deterioration process [8]. Furthermore, cutting-edge research has expanded into the realms of multi-field coupling and mesoscopic simulation. Multi-field coupling constitutive models integrate humidity, chemical, and mechanical fields to elucidate hydro-mechanical, chemo-mechanical, and fully coupled degradation mechanisms. At the mesoscale, discrete element methods and random aggregate models, in conjunction with advanced observational techniques such as CT scanning and SEM, have been employed to investigate the localized degradation behavior of aggregates, mortar matrices, and interfacial transition zones. These mesoscopic investigations provide a physical basis for macroscopic constitutive models [9,10,11]. Concurrently, the scope of this research has broadened to encompass coupled corrosion-load conditions and innovative material systems, including recycled aggregate concrete and geopolymer concrete.

Recent studies have further advanced constitutive modeling toward multi-field coupling and data-driven frameworks. Meng et al. investigated the chemo-mechanical damage evolution of concrete under the combined action of sulfate attack and cyclic loading, highlighting the interaction between environmental deterioration and mechanical damage. Zhang et al. subsequently developed a hydro-chemo-mechanical coupled model for unsaturated concrete subjected to sulfate attack, moisture cycling, and mechanical loading. In parallel, data-driven approaches have increasingly been introduced into constitutive modeling; for example, Zhang et al. developed a data-driven rate-dependent constitutive model for ultra-high-strength alkali-activated concrete, demonstrating the potential of data-driven frameworks for reproducing complex stress–strain responses [12,13,14].

Despite the substantial body of existing research, the limited engineering applicability of current models remains a critical constraint. First, the stress states and environmental conditions imposed on laboratory specimens often fail to accurately replicate the complex realities of in-service structures. Second, pronounced scale effects between small-scale specimens and full-scale structural components lead to significant deviations in observed damage evolution behavior. Third, the long-term predictive capabilities of existing models remain inadequate; consequently, their incorporation into mainstream design codes has been sparse, limiting their wider application in engineering practice [15,16,17]. Moving forward, research must focus on advancing the quantification of microscopic degradation mechanisms, refining multi-field coupling models, standardizing experimental testing protocols, and facilitating direct field implementation. Addressing these areas will drive the development of concrete constitutive models subjected to wetting–drying cycles toward greater accuracy, reliability, and practical engineering utility [18,19,20].

To address these gaps, this study focuses on concrete located within the wetting–drying zones of in-service bridge piers. Grounded in the strain equivalence principle and the Weibull statistical distribution theory, a novel uniaxial compressive constitutive model is established that explicitly incorporates the influence of saturated porosity. The engineering applicability of the proposed model is subsequently validated through both controlled laboratory testing and field applications on an actual bridge structure. Ultimately, this research provides theoretical support for the performance evaluation and structural strengthening of concrete infrastructure subjected to repeated wetting–drying exposure.

## 2. Formulation of the Porosity-Dependent Constitutive Model

**Figure 1:**
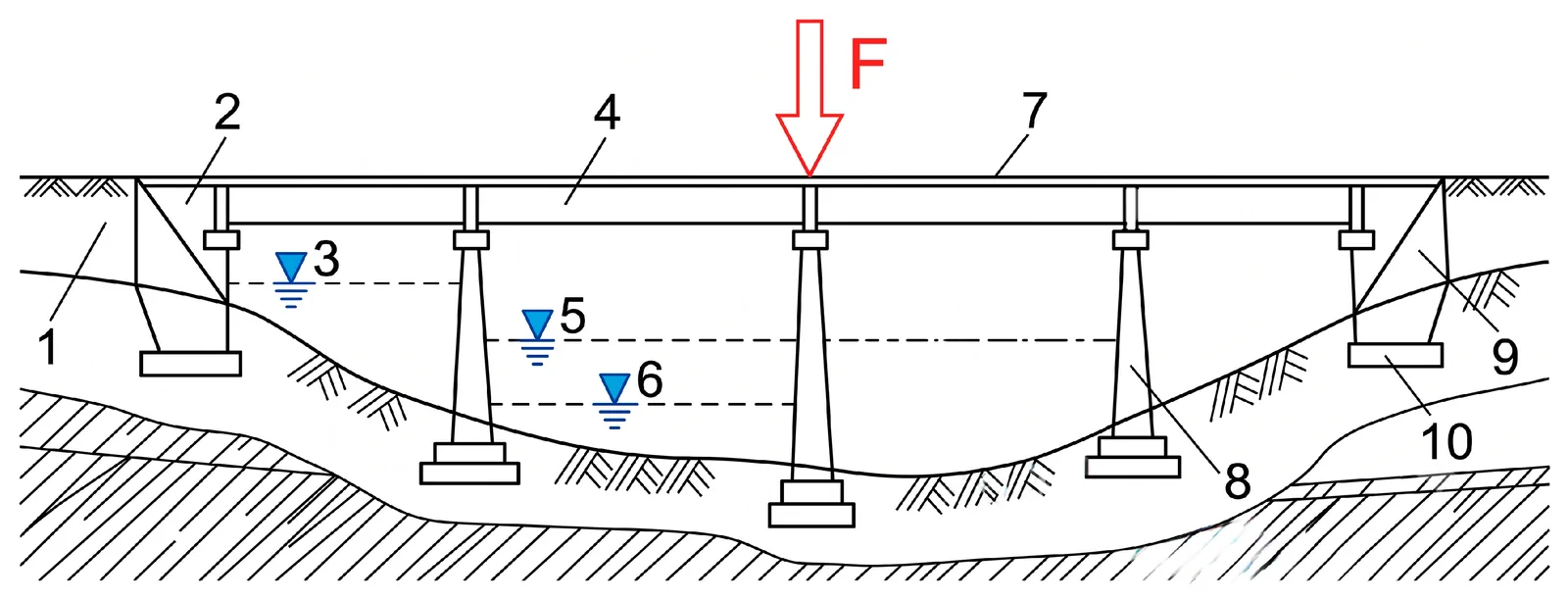
Schematic representation of an in-service bridge pier subjected to uniaxial compression.

In-service bridge piers are continuously subjected to long-term vertical compression; thus, their structural stress state can be reasonably approximated as uniaxial compression combined with exposure to wetting–drying cycles (Figure 1). Grounded in the strain equivalence principle, the constitutive relationship of a porous material under uniaxial stress can be mathematically derived from that of a continuous medium. This derivation is obtained by substituting the nominal stress in the standard continuum formulation with an effective stress that explicitly accounts for the saturated porosity of the material. As illustrated in Figure 1, the schematic details the structural components and environmental conditions of an in-service bridge subjected to varying hydrological states. Specifically, components 1 and 9 represent the riverbank subgrade, while component 2 denotes the bridge abutment. The bridge superstructure consists of the main girder (4) and the bridge deck (7). The substructure includes the main load-bearing bridge piers (8) and their underlying foundations (10). The hydrological environment is characterized by fluctuating water elevations, where markers 3, 5, and 6 indicate the flood-season high water level, the intermediate fluctuating water level, and the dry-season low water level, respectively. The vertical region bounded by the high and low water levels (between markers 3 and 6) defines the critical wetting–drying fluctuation zone where the pier concrete is continuously subjected to severe cyclic environmental exposure. Additionally, the red arrow labeled ’F’ represents the long-term vertical compressive load transferred from the superstructure to the piers, which establishes the uniaxial compression state evaluated in this study.

**Figure 2:**
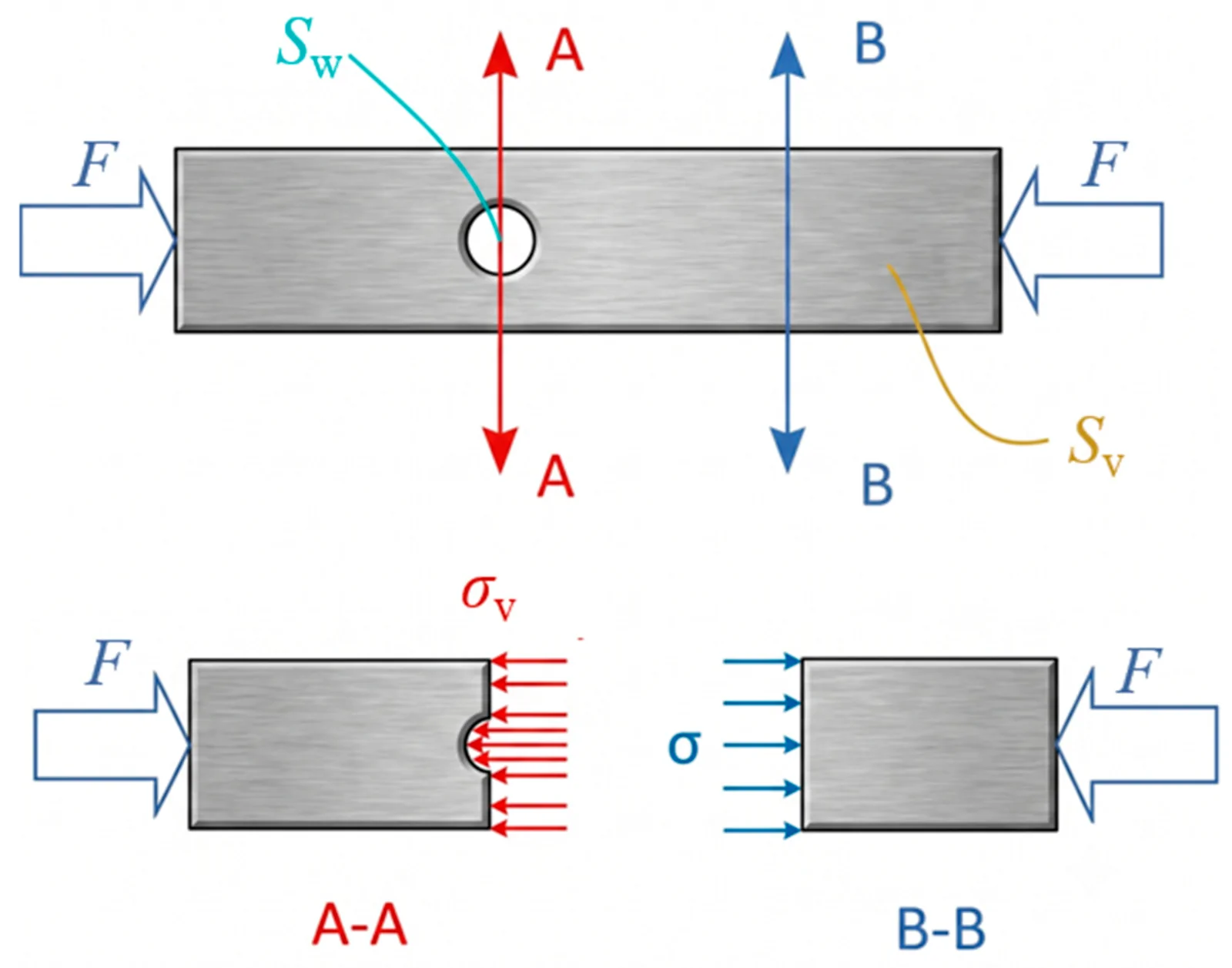
Schematic diagram of the stress distribution in porous concrete.

In Figure 2, let *S*_V_ denote the solid volume that sustains the applied load in the presence of internal voids, *S*_W_ represent the saturated pore volume, and *S* be the total volume of the saturated concrete under natural conditions. Following the fundamental volumetric relationship *S* = *S*_V_ + *S*_W_, the initial saturated porosity *P*_0_ is defined as *P*_0_ = *S*_W_/*S* = *S*_W_/(*S*_W_ + *S*_V_), where *P*_0_ is dimensionless and represents the experimentally measurable pore state of the concrete prior to mechanical loading.

According to the fundamental principles of solid mechanics, the constitutive equation for dense concrete is expressed as follows:

(1) $$\sigma_{v}=E\varepsilon$$

where *σ*_v_ represents the stress within the dense solid matrix of the porous concrete, *E* denotes the elastic modulus of the material, and *ε* is the corresponding strain.

To characterize the progressive reduction in effective load-bearing capacity during compression, a strain-dependent pore-related statistical damage variable *D*(*ε*) is introduced. The nominal stress is expressed as:

(2) $$\sigma =\sigma_{v}\left[1-D\left(\varepsilon \right)\right]$$

where *σ* represents the nominal stress and *D*(*ε*) denotes the strain-dependent statistical damage variable.

The factor [1 − *D*(*ε*)] represents an idealized effective load-bearing fraction associated with progressive material damage. It should be emphasized that Equation (2) is not an empirical linear regression between the experimentally measured porosity and compressive stress. The measured initial saturated porosity *P*_0_ is treated separately and is introduced later in the porosity correction. In addition, pore size, morphology, connectivity, spatial distribution, and interactions with microcracks are not explicitly resolved in this simplified macroscopic formulation.

Substituting Equation (1) into Equation (2) and rearranging the terms yields:

(3) $$\sigma =E\varepsilon \left[1-D\left(\varepsilon \right)\right]$$

Concrete is a heterogeneous quasi-brittle material containing pores, microcracks, aggregates, cement paste, and interfacial transition zones; therefore, the failure thresholds of its microscopic load-bearing elements exhibit statistical variability. Previous studies have reported that pore size and pore diameter distributions in pervious or porous concrete can be reasonably characterized using Weibull-type statistical distributions [21,22]. These observations provide a statistical basis for describing pore-related heterogeneity, but they do not imply that the experimentally measured initial saturated porosity *P*_0_ itself follows a Weibull distribution.

Accordingly, a two-parameter Weibull cumulative distribution function is introduced to describe the evolution of the strain-dependent statistical damage variable *D*(*ε*):

(4) $$D\left(\varepsilon \right)=1-\exp\left[-{\left(\frac{\varepsilon }{\beta }\right)}^{\lambda }\right]$$

where *λ* denotes the shape parameter and *β* represents the scale parameter.

In accordance with the governing principles of continuum mechanics, the axial stress–strain relationship of porous concrete subjected to uniaxial compression is mathematically described by Equation (3). Substituting Equation (4) into Equation (3) yields:

(5) $$\sigma =E\varepsilon \exp\left[-{\left(\frac{\varepsilon }{\beta }\right)}^{\lambda }\right]$$

**Figure 3:**
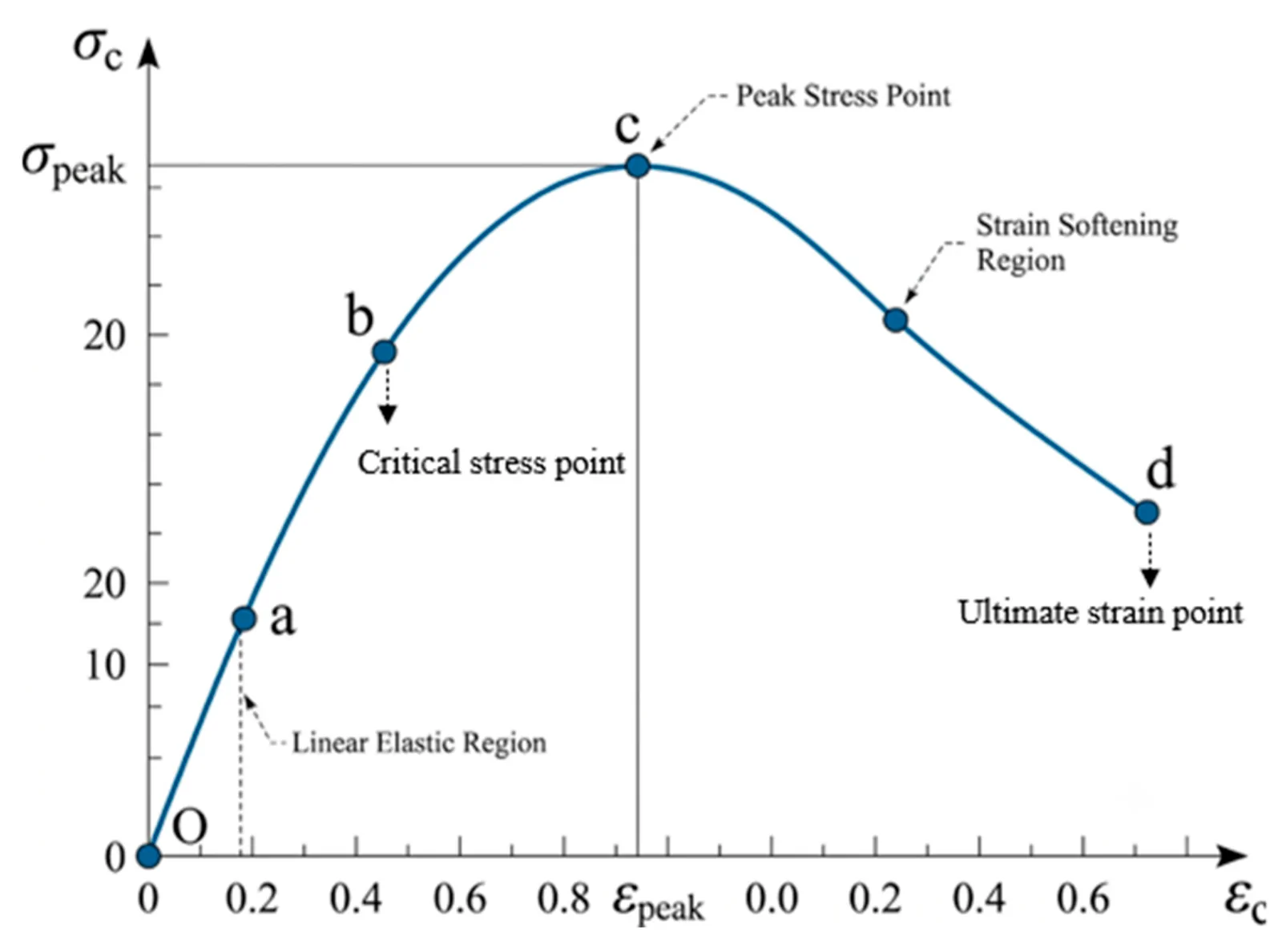
Stress–strain curve of concrete.

Based on the geometric characteristics of the complete stress–strain curve for concrete (as illustrated in Figure 3), the fundamental boundary conditions for the porous concrete material can be mathematically established. Specifically, the four essential criteria are defined as follows: Boundary Condition 1 corresponds to the origin point where the strain and stress are zero ($\sigma_{0}=0,\;\varepsilon_{0}=0$); Boundary Condition 2 represents the initial elastic tangent at the origin ($\frac{d\sigma_{0}=0}{d\varepsilon_{0}=0}=E$); Boundary Condition 3 identifies the peak coordinate where the material reaches its maximum capacity ($\sigma_{c}=\sigma_{pk},\;\varepsilon_{c}=\varepsilon_{pk}$); and Boundary Condition 4 dictates that the slope of the curve is zero at this peak stress point ($\frac{d\sigma_{c}}{d\varepsilon_{c}}=0$). where *σ_pk_* and *ε_pk_* represent the peak stress and peak strain at point c, respectively, while *E* denotes the initial elastic modulus of the concrete.

Differentiating Equation (5) with respect to the strain *ε* yields:

(6) $$\frac{d\sigma }{d\varepsilon }=E\exp\left[-{\left(\frac{\varepsilon }{\beta }\right)}^{\lambda }\right]\left[1-\lambda {\left(\frac{\varepsilon }{\beta }\right)}^{\lambda }\right]$$

Applying the boundary condition at point c (corresponding to the peak stress) yields:

(7) $$0=E\exp\left[-{\left(\frac{\varepsilon_{pk}}{\beta }\right)}^{\lambda }\right]\left[1-\lambda {\left(\frac{\varepsilon_{pk}}{\beta }\right)}^{\lambda }\right]$$

Based on the fundamental mechanical properties of concrete, the initial elastic modulus is strictly non-zero (*E* ≠ 0), and the associated exponential term is inherently positive (i.e., $\exp\left[-{\left(\frac{\varepsilon_{pk}}{\beta }\right)}^{\lambda }\right]\neq 0$). Consequently, the relationship simplifies, yielding:

(8) $$\left[1-\lambda {\left(\frac{\varepsilon_{pk}}{\beta }\right)}^{\lambda }\right]=0$$

Rearranging the terms of Equation (8) yields:

(9) $${\left(\frac{\varepsilon_{pk}}{\beta }\right)}^{\lambda }=\frac{1}{\lambda }$$

Taking both sides of Equation (9) to the power 1/*λ* and rearranging yields:

(10) $$\beta =\varepsilon_{pk}\lambda^{\frac{1}{\lambda }}$$

Substituting Equation (10) into Equation (5) and applying Boundary Condition 3 at the peak point ($\varepsilon_{pk}$,$\sigma_{pk}$), the following expression is obtained:

(11) $$\lambda =\frac{1}{\ln\frac{E\varepsilon_{pk}}{\sigma_{pk}}}$$

Substituting Equation (10) into Equation (4) and rearranging the terms yields:

(12) $$D\left(\varepsilon \right)=1-\exp\left(-\frac{1}{\lambda }{\left(\frac{\varepsilon }{\varepsilon_{pk}}\right)}^{\lambda }\right)$$

Based on Equations (11) and (12), the strain-dependent pore-related statistical damage variable *D*(*ε*) is governed by the peak strain, peak stress, initial elastic modulus, and instantaneous strain. For a given concrete mixture, E, *σ_pk_*, and *ε_pk_* are treated as calibrated material parameters, whereas *D*(*ε*) evolves progressively with increasing strain. Consequently, substituting Equations (12) into Equations (3) yields:

(13) $$\sigma =E\varepsilon \exp\left(-\frac{1}{\lambda }{\left(\frac{\varepsilon }{\varepsilon_{pk}}\right)}^{\lambda }\right)$$

Equation (13) describes the nominal stress–strain response of concrete considering the progressive statistical damage of the effective load-bearing structure during compression. At this stage, *D*(*ε*) represents loading-induced statistical damage and should be distinguished from the experimentally measured initial saturated porosity *P*_0_.

To incorporate the influence of the experimentally measured initial saturated porosity $P_{0}$, the porosity-corrected effective stress is defined as $\sigma_{eff}=\sigma (1-P_{0})$. Substituting Equation (13) into the above porosity-correction relationship yields:

(14) $$\sigma_{\mathrm{eff}}=\frac{E\varepsilon }{1-P}\exp\left(-\frac{1}{\lambda }{\left(\frac{\varepsilon }{\varepsilon_{pk}}\right)}^{\lambda }\right)$$

Equation (14) combines the measured initial pore state *P*_0_ with the strain-dependent statistical damage evolution *D*(*ε*). Here, *P*_0_ characterizes the initial pore condition of the specimen, whereas *D*(*ε*) characterizes the progressive damage associated with mechanical loading. Equation (14) should therefore be interpreted as the porosity-corrected effective-stress representation corresponding to the nominal constitutive relationship of Equation (13), rather than as an independent constitutive law.

## 3. Experimental Validation

### 3.1. Material Specifications and Mix Proportions

To empirically validate the proposed constitutive model, concrete specimens with varying porosities were fabricated and subjected to uniaxial compression tests. The mix proportioning of pervious concrete requires a balance of multiple factors, including permeability, mechanical properties, and constructability. In this study, the concrete mixtures were formulated using coarse aggregate, cement, water, and specialized admixtures (such as cementing and reinforcing agents). Given its significant influence on both hydraulic and structural behavior, porosity serves as a fundamental parameter for evaluating the overall performance of the pervious concrete.

In this study, P·O 42.5 ordinary Portland cement was utilized, comprising 30% of the total solid volume. To facilitate the desired void structure, the proportion of fine aggregate in the mix design was intentionally minimized. The coarse aggregate framework consisted of basalt manufactured sand and continuously graded crushed stone with a particle size range of 5–10 mm, altogether accounting for 65% of the total volume. The water-to-cement mass ratio (*w*/*c*) was strictly controlled between 0.3 and 0.4 to ensure an appropriate balance of mixture workability and final permeability. Additionally, a tailored dosage of a specialized SR cementing agent was incorporated to improve mechanical strength and interfacial cohesion without compromising the target high porosity. Finally, steel fibers were introduced into the cementitious matrix to augment the concrete’s overall crack resistance. To systematically investigate the influence of the void structure, three target porosity levels—15%, 20%, and 25%—were established for this study, resulting in the formulation of six distinct mix designs. The detailed material proportions for each pervious concrete mixture are summarized in Table 1.

The specific materials, reagents, equipment, and software utilized in this study are detailed as follows. The P·O 42.5 ordinary Portland cement was sourced from Anhui Conch Cement Co., Ltd., Wuhu, China. The SR cementing agent and steel fibers were supplied by Sobute New Materials Co., Ltd., Nanjing, China and Ganzhou Daye Metallic Fibres Co., Ltd., Ganzhou, China. For the wetting–drying exposure tests, sodium sulfate (Na2SO4) and the standard phenolphthalein indicator solution used for carbonation depth measurements were purchased from Sinopharm Chemical Reagent Co., Ltd., Shanghai, China and Shanghai Macklin Biochemical Technology Co., Ltd., Shanghai, China. Macroscopic physical and mechanical evaluations were conducted using a specialized permeability apparatus (Model HS-4) manufactured by Beijing Jianyi Experiment Instrument Co., Ltd., Beijing, China and a universal testing machine (Model WAW-1000D) provided by Jinan Shidai Shijin Testing Machine Co., Ltd., Jinan, China. For the field investigations at the Saiqi Bridge, a moisture testing apparatus (Model HK-30, Taizhou Huake Instrument Co., Ltd., Taizhou, China), a rebound hammer (Model HT-225, Beijing ZBL Science and Technology Co., Ltd., Beijing, China), and an impact drill (Model Z1C-FF05-26, Jiangsu Dongcheng Power Tools Co., Ltd., Nantong, China) were employed. Finally, all data processing, statistical analysis, and theoretical curve fitting were performed using Origin software, version 2021 (OriginLab Corporation, Northampton, MA, USA).

### 3.2. Testing Methodology and Wetting–Drying Exposure

Following standard curing for 28 days in a curing room maintained at 20 ± 2 °C and a relative humidity above 95%, the effective porosity of the 15 × 15 × 15 cm cubic pervious concrete specimens was determined using the water-saturation method based on the dry, submerged, and saturated surface-dry masses, with the same weighing and calculation procedure applied to all specimen groups. The measured porosity results for each mixture are detailed in Table 2. Subsequently, to simulate aggressive environmental degradation, the specimens were subjected to accelerated wetting–drying cycles in a 10% sodium sulfate (Na_2_SO_4_) solution.

Each 30-day exposure interval consisted of two successive wetting–drying alternations. Specifically, the specimens were first immersed in the 10% Na_2_SO_4_ solution for 8 days and subsequently dried at room temperature for 8 days, followed by a second immersion period of 7 days and a final ambient drying period of 7 days. Thus, the cumulative wetting and drying durations within each 30-day interval were both 15 days, maintaining an overall wetting-to-drying duration ratio of 1:1.

This exposure sequence was adopted to maintain an equal cumulative wetting-to-drying duration within each 30-day observation interval while providing two repeated wetting–drying alternations. Previous studies have shown that the dry–wet duration ratio and cycle period can significantly influence sulfate-induced deterioration, and a 1:1 wetting-to-drying ratio has been employed in previous sulfate-exposure studies [23,24]. The slight difference between the first 8-day and second 7-day stages was introduced solely to accommodate two successive alternations within the fixed 30-day testing interval and does not represent two different environmental exposure conditions.

The duration of the exposure program was selected to provide an extended observation window for the progressive deterioration of concrete under sulfate wetting–drying conditions. Previous studies have demonstrated that the duration and number of wetting–drying cycles significantly affect sulfate-induced deterioration, and that concrete properties may exhibit different evolution stages as exposure accumulates [7,23]. Therefore, 15 consecutive 30-day exposure intervals, corresponding to a total exposure duration of 450 days, were adopted in the present study to capture both the early-stage evolution and the subsequent deterioration of the physical and mechanical properties. It should be emphasized that the 450-day laboratory exposure was designed as a controlled long-term accelerated deterioration program and does not represent a direct time equivalence to the 25-year service period of the Saiqi Bridge.

All experimental procedures were conducted in strict accordance with the specifications outlined in GB/T 50082-2024 [25] (Standard for Test Methods of Long-term Performance and Durability of Concrete) and CECS 13 [26] (Standard for Test Methods of Fiber Reinforced Concrete).

For international methodological comparison and engineering applicability, relevant international standards and recommendations are also cited as methodological references. ISO 17785-1 [27] provides a laboratory method for determining the infiltration rate of hardened pervious concrete, while ISO 17785-2 [28] specifies methods for determining the density and void content of pervious concrete. ASTM C1701/C1701M [29] provides a field method for evaluating the infiltration rate of in-place pervious concrete, and the RILEM TC 116-PCD [30] recommendations provide methodological guidance for permeability and capillary water absorption testing of hardened concrete. These documents are cited for international methodological comparison rather than as the governing procedures of the present experimental program.

The permeability coefficient of each specimen was determined utilizing a specialized permeability apparatus.

**Figure 4:**
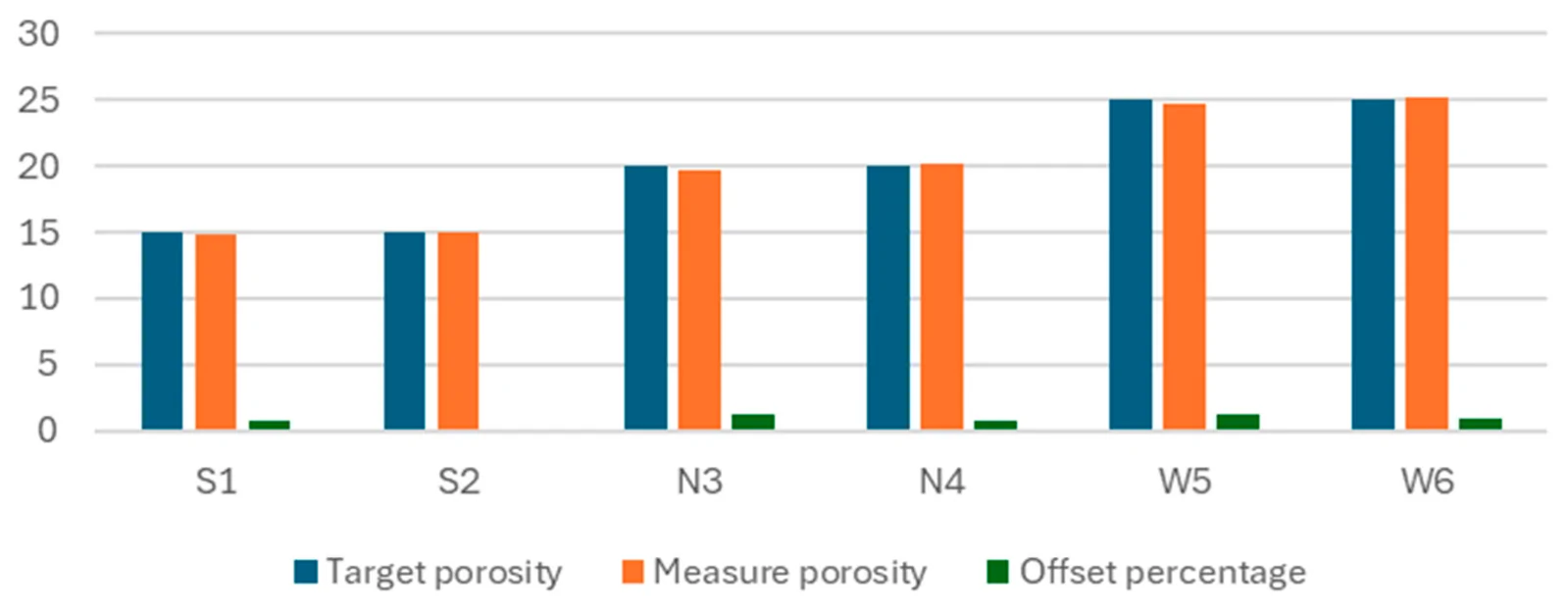
Comparison between target and measured porosities for the pervious concrete specimens.

The measured porosity results for the six specimen groups are graphically presented in Figure 4. As depicted, the empirical values align closely with the designed target porosities, with all deviations strictly confined to a 2% margin. Notably, the magnitude of the measurement error exhibits a proportional increase as the target porosity rises. Conversely, higher compressive strength in the concrete matrix is consistently associated with minimized measurement discrepancies. This suggests that enhanced mechanical strength may contribute to the dimensional stability of the internal pore network.

### 3.3. Analysis of Test Results

**Figure 5:**
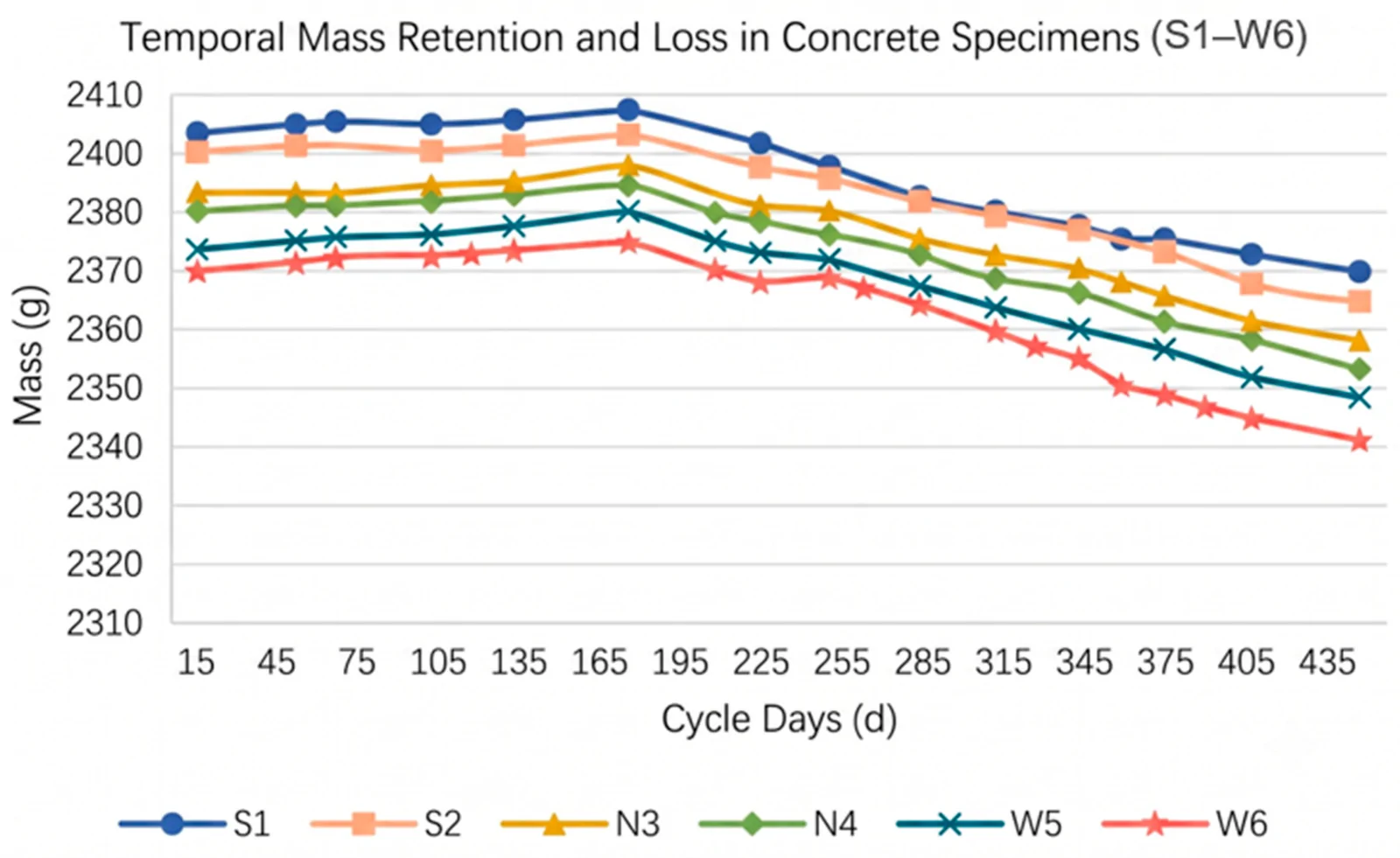
Evolution of specimen mass over successive sulfate wetting–drying cycles.

To quantify the physical degradation induced by the environmental exposure, the mass of the specimens was measured following each wetting–drying cycle and evaluated relative to their initial pre-exposure baseline. The progressive variation in specimen mass as a function of exposure time for all mixture groups is graphically illustrated in Figure 5.

During the cyclic sulfate wetting–drying exposure, the mass evolution of all specimens followed a distinct biphasic pattern characterized by an initial period of mass gain followed by a subsequent gradual decrease. Notably, specimens with lower porosity exhibited more pronounced peak mass gains, indicating an inverse relationship between the designed porosity and the overall magnitude of mass variation. Furthermore, the absence of any precipitous mass loss or sudden spalling across all specimen groups throughout the extended cyclic exposure demonstrates that the pervious concrete maintained structural integrity and possesses relatively good resistance to sulfate-induced degradation.

The permeability coefficients of the six specimen groups were determined utilizing a specialized permeability apparatus, with the empirical results summarized in Table 2. Analysis of the data reveals that the permeability coefficient exhibits a progressive increase as the number of wetting–drying cycles accumulates. As anticipated, a higher designed porosity inherently correlates with a larger overall permeability. Furthermore, the magnitude of this cyclic permeability enhancement becomes significantly more pronounced under extended environmental exposure.

At the same target porosity, the incorporation of the SR binder and steel fibers resulted in only a limited change in hydraulic performance. Compared with the corresponding ordinary mixtures, the average permeability coefficients of S2, N4, and W6 increased by approximately 3.2%, 4.4%, and 1.9%, respectively. Meanwhile, the reinforced mixtures also exhibited slightly higher measured porosities than their corresponding ordinary mixtures. Therefore, the observed differences in permeability cannot be attributed independently to the SR binder and steel fibers. Instead, the present results indicate that the overall hydraulic performance is governed predominantly by the pore structure associated with the designed porosity, whereas the principal functions of the SR binder and steel fibers in the present mix design are to improve matrix cohesion, mechanical integrity, and crack resistance while maintaining the required permeable pore network. Accordingly, the hydraulic benefit of these reinforcing components should not be overstated.

Using the measured porosity together with the calibrated mechanical parameters obtained from the uniaxial compression response, the group-specific constitutive relationships were derived. The permeability coefficient and mass variation were used as complementary indicators of physical deterioration rather than as direct input variables of Equation (14). The calibrated model parameters and the resulting group-specific constitutive equations are summarized in Table 3. It is crucial to clarify the physical significance of the parameter *E*_0_ within this analytical framework. Based on the strain equivalence principle defined in Equation (1), *E*_0_ denotes the theoretical intrinsic elastic modulus of the dense solid matrix, rather than the macroscopic apparent elastic modulus of the bulk porous concrete. Because the macro-scale structural weakening induced by the void volume is explicitly decoupled and independently governed by the porosity-correction factor (1 − *P*_0_) in Equation (14), and given that the water-to-cement ratio (0.3–0.4) and aggregate constituents were maintained consistently across all mixtures, the intrinsic physical properties of the solid skeleton are assumed to remain uniform. Consequently, *E*_0_ is treated as a material constant across the different porosity groups to isolate the mechanical influence of the experimentally measured porosity *P*_0_.

**Figure 6:**
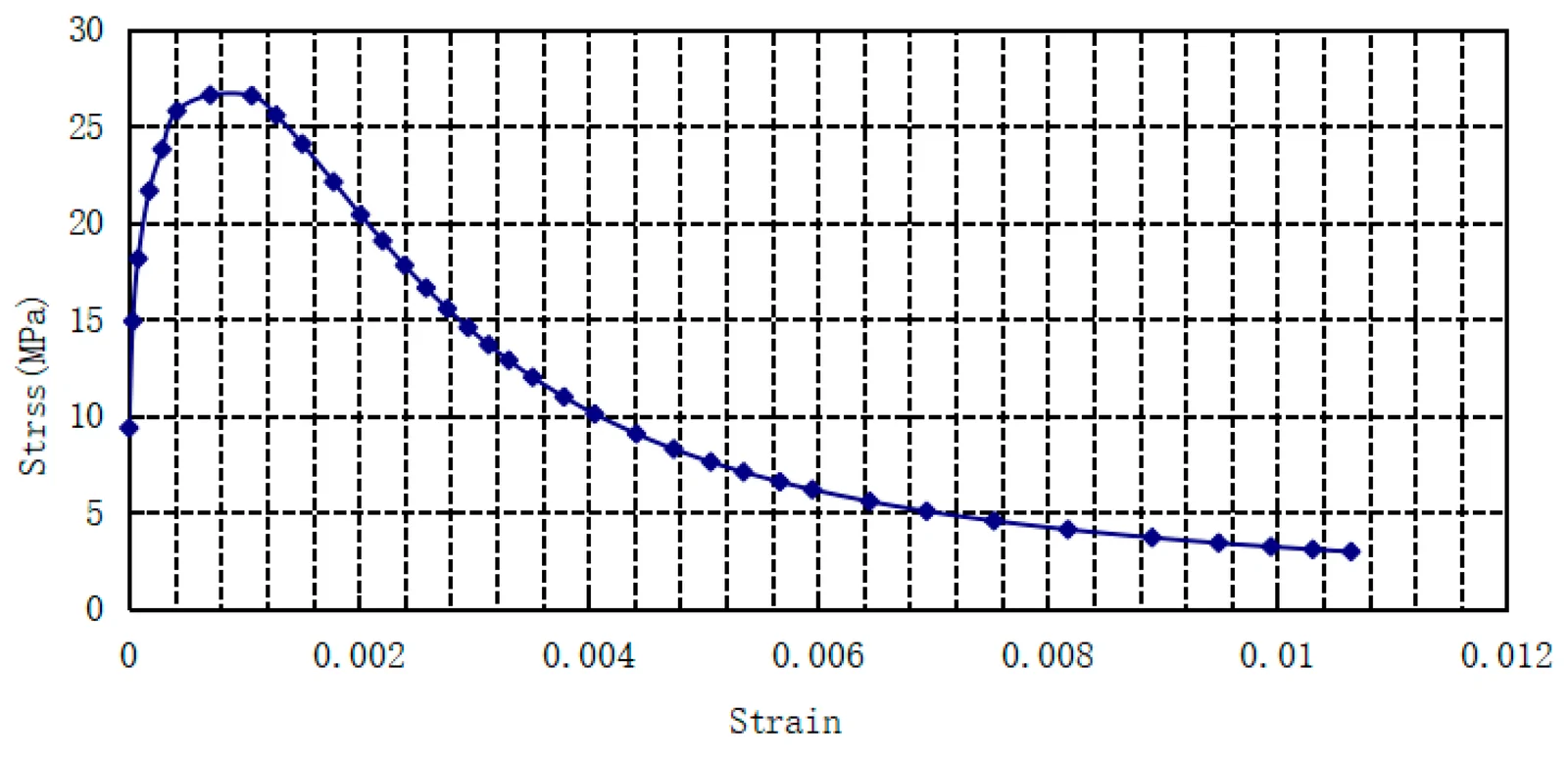
Comparison of experimental and theoretical uniaxial compressive stress–strain curves for Group S1.

**Figure 7:**
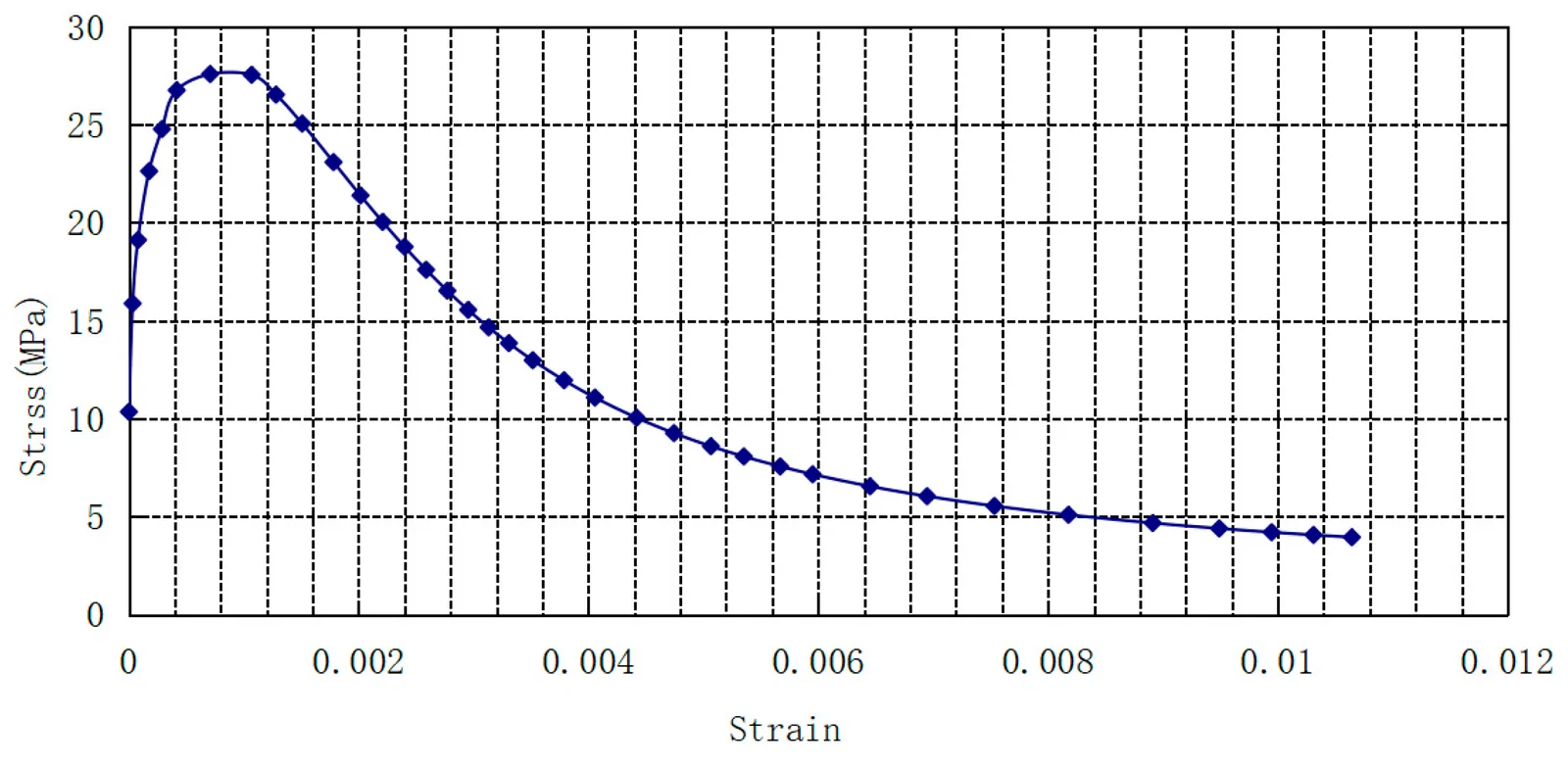
Comparison of experimental and theoretical uniaxial compressive stress–strain curves for Group S2.

**Figure 8:**
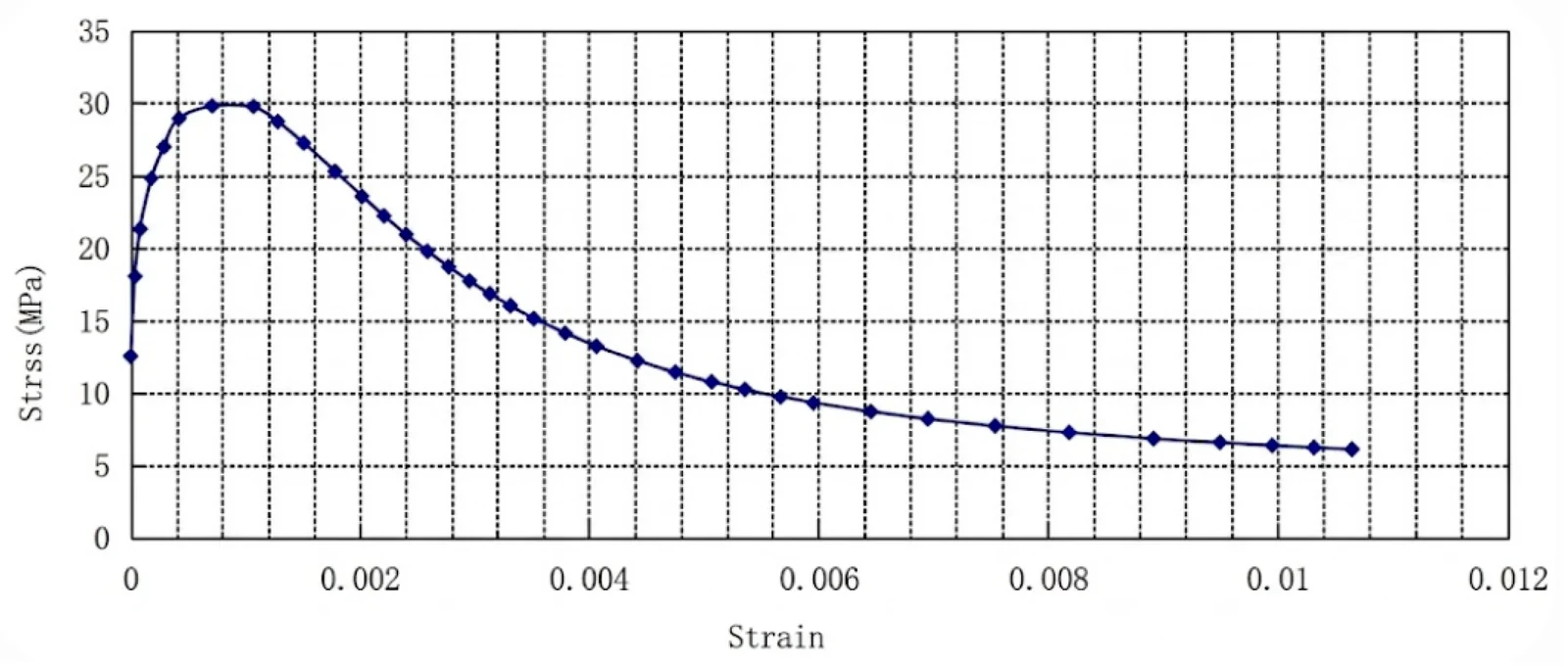
Comparison of experimental and theoretical uniaxial compressive stress–strain curves for Group N3.

**Figure 9:**
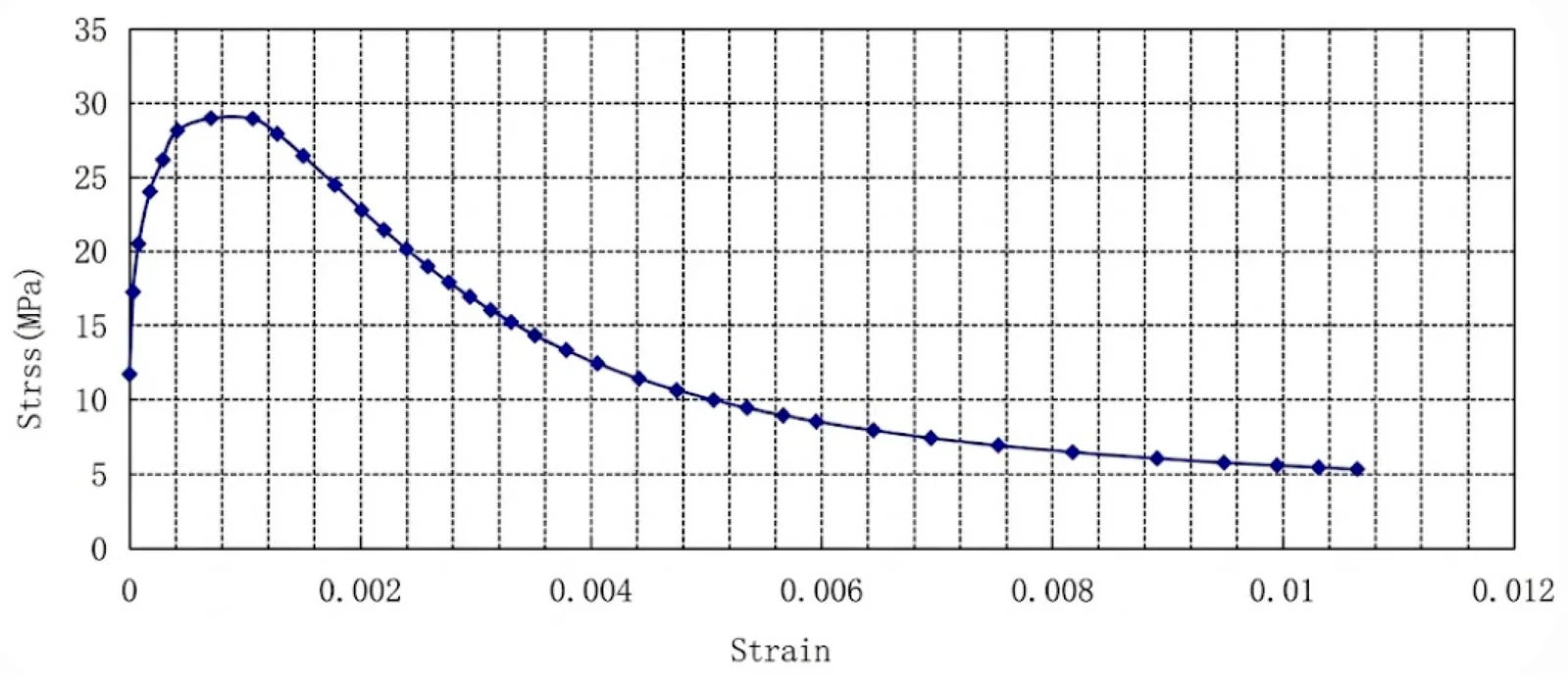
Comparison of experimental and theoretical uniaxial compressive stress–strain curves for Group N4.

**Figure 10:**
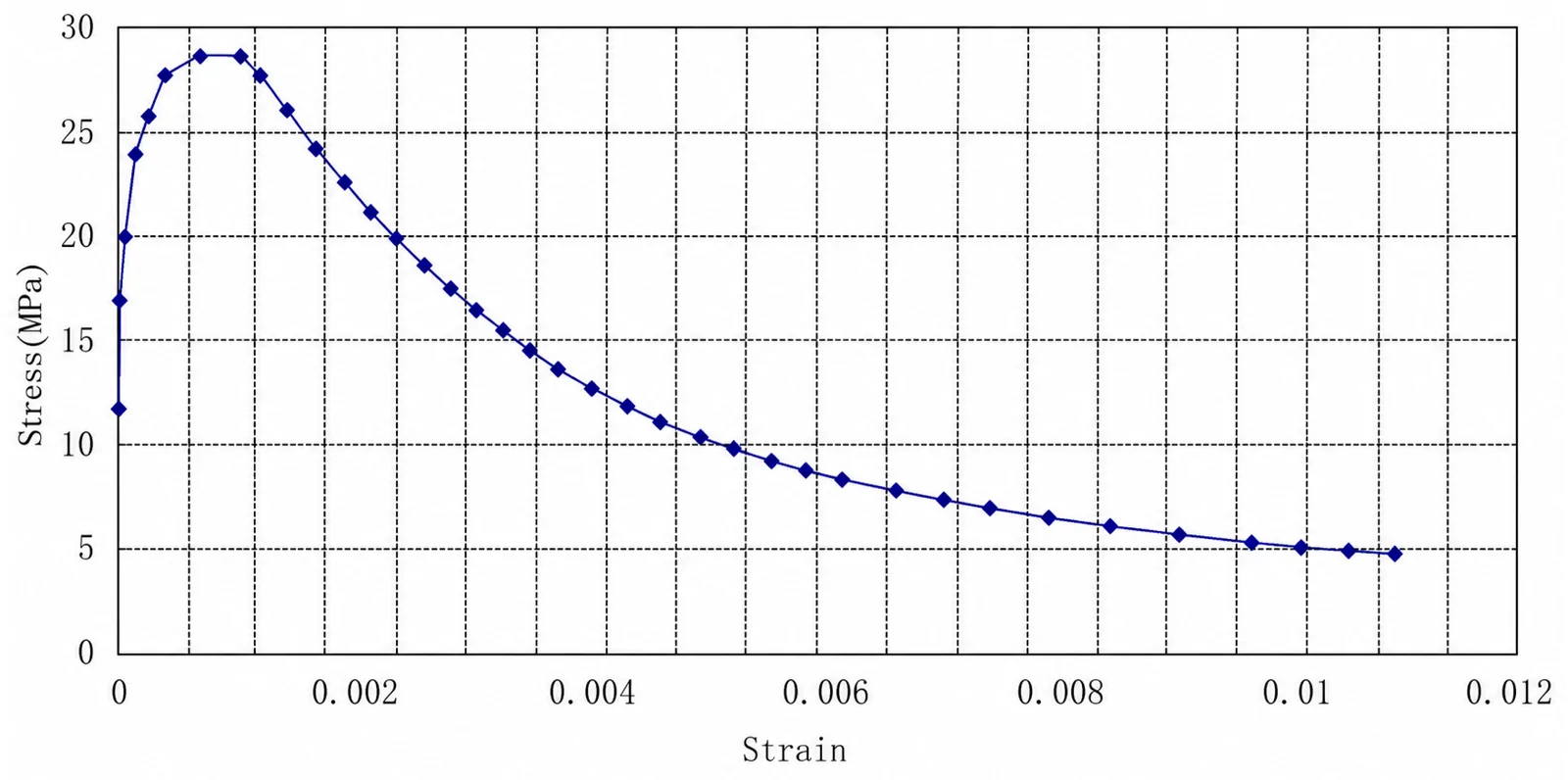
Comparison of experimental and theoretical uniaxial compressive stress–strain curves for Group W5.

**Figure 11:**
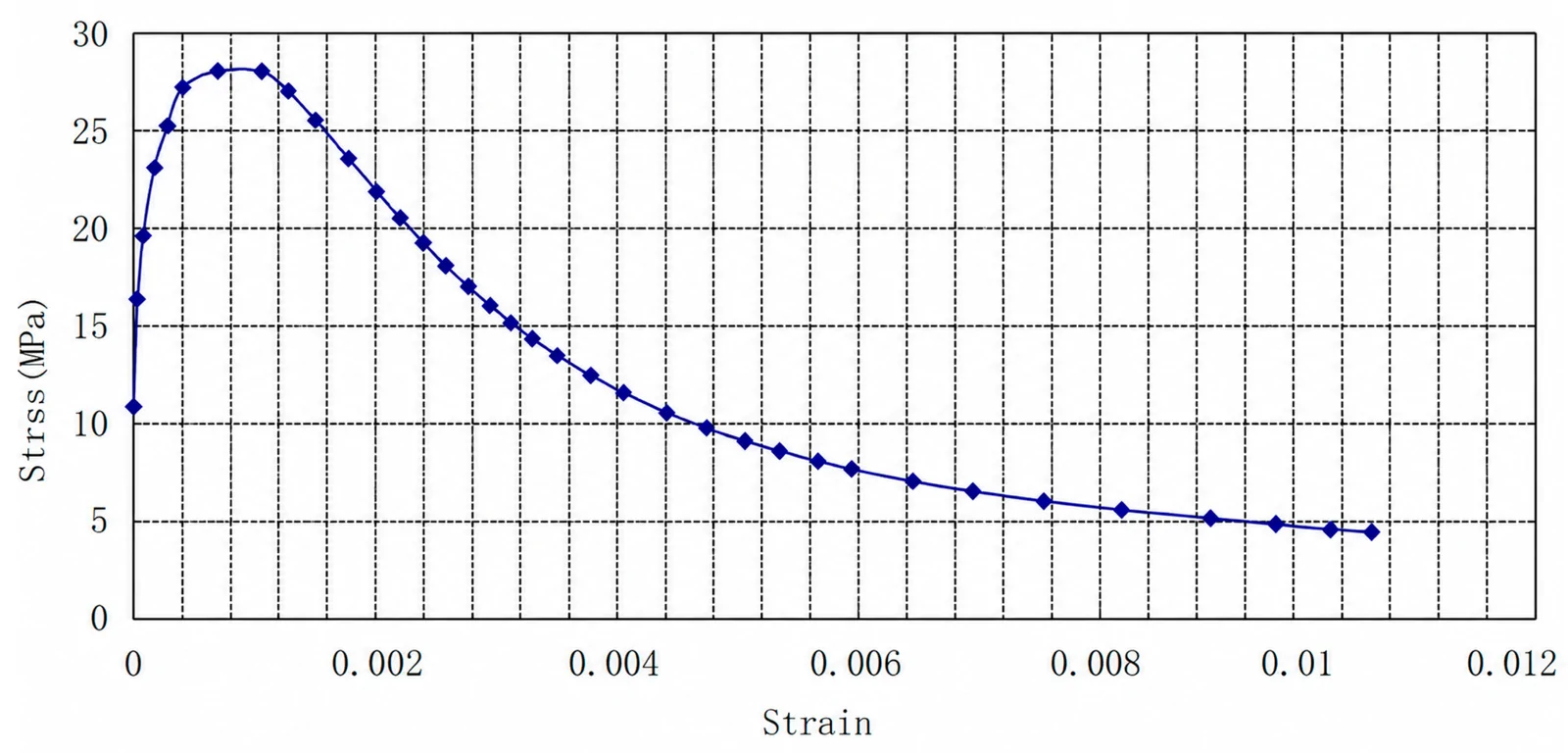
Comparison of experimental and theoretical uniaxial compressive stress–strain curves for Group W6.

Uniaxial compression tests were subsequently executed on all six specimen groups to evaluate their macroscopic mechanical response. A comparative analysis between the empirically measured stress–strain curves and the theoretically predicted constitutive relationships is graphically presented in Figure 6, Figure 7, Figure 8, Figure 9, Figure 10 and Figure 11.

As evidenced by the comparative stress–strain profiles, the theoretical predictions generated by the proposed constitutive model exhibit a good agreement with the experimental results. This close agreement validates the model’s capability to describe the complex mechanical behavior of concrete subjected to cyclic wetting–drying exposure. Consequently, the established analytical formulation provides a useful framework for the numerical simulation and structural assessment of in-service concrete infrastructure operating under similar environmental degradation.

To further quantify the agreement between the theoretical predictions and the experimental results, the experimental stress–strain data points presented in Figure 6, Figure 7, Figure 8, Figure 9, Figure 10 and Figure 11 were digitized, and the root mean square error (RMSE) and coefficient of determination (R^2^) were calculated for each specimen group. The quantitative comparison results are summarized in Table 4.

As shown in Table 4, the R^2^ values range from 0.9966 to 0.9998, while the RMSE values range from 0.101 to 0.456 MPa. These quantitative indices indicate very good agreement between the proposed constitutive model and the experimental stress–strain responses for all six specimen groups. Although some variation in fitting accuracy (RMSE ranging from 0.101 to 0.456 MPa) is observed among the different mixtures, the overall prediction accuracy remains consistently high within the investigated porosity range. The slight fluctuations in predictive accuracy can be primarily attributed to the inherent material heterogeneity of pervious concrete. Specifically, for specimens with higher target porosities (e.g., W5 and W6), the highly randomized spatial distribution of coarse aggregates and irregular pore networks intensify localized stress concentrations and introduce stochastic noise into the macroscopic stress–strain response. Nevertheless, the consistently high R^2^ values (>0.996) confirm the general robustness of the proposed framework.

The present calibration and experimental validation mainly concern the ascending branch of the stress–strain response up to the peak stress. Therefore, application of the model to the post-peak descending branch requires additional calibration and independent experimental validation.

It should be noted that the laboratory validation in the present study was conducted using pervious concrete with target porosities of 15%, 20%, and 25%. Therefore, the experimentally validated applicability of the proposed model is currently limited to this relatively high-porosity range. Although the mathematical formulation can accommodate lower porosity values, conventional structural concrete generally possesses a denser pore structure, and its pore connectivity, elastic response, and deterioration characteristics may differ from those of the pervious concrete investigated herein. Consequently, direct quantitative application of the present model to lower-porosity conventional structural concrete should be performed with caution and requires further calibration and experimental validation. Future studies will extend the experimental database to lower porosity ranges representative of conventional structural concrete.

## 4. Field Application and Case Study: Saiqi Bridge

**Figure 12:**
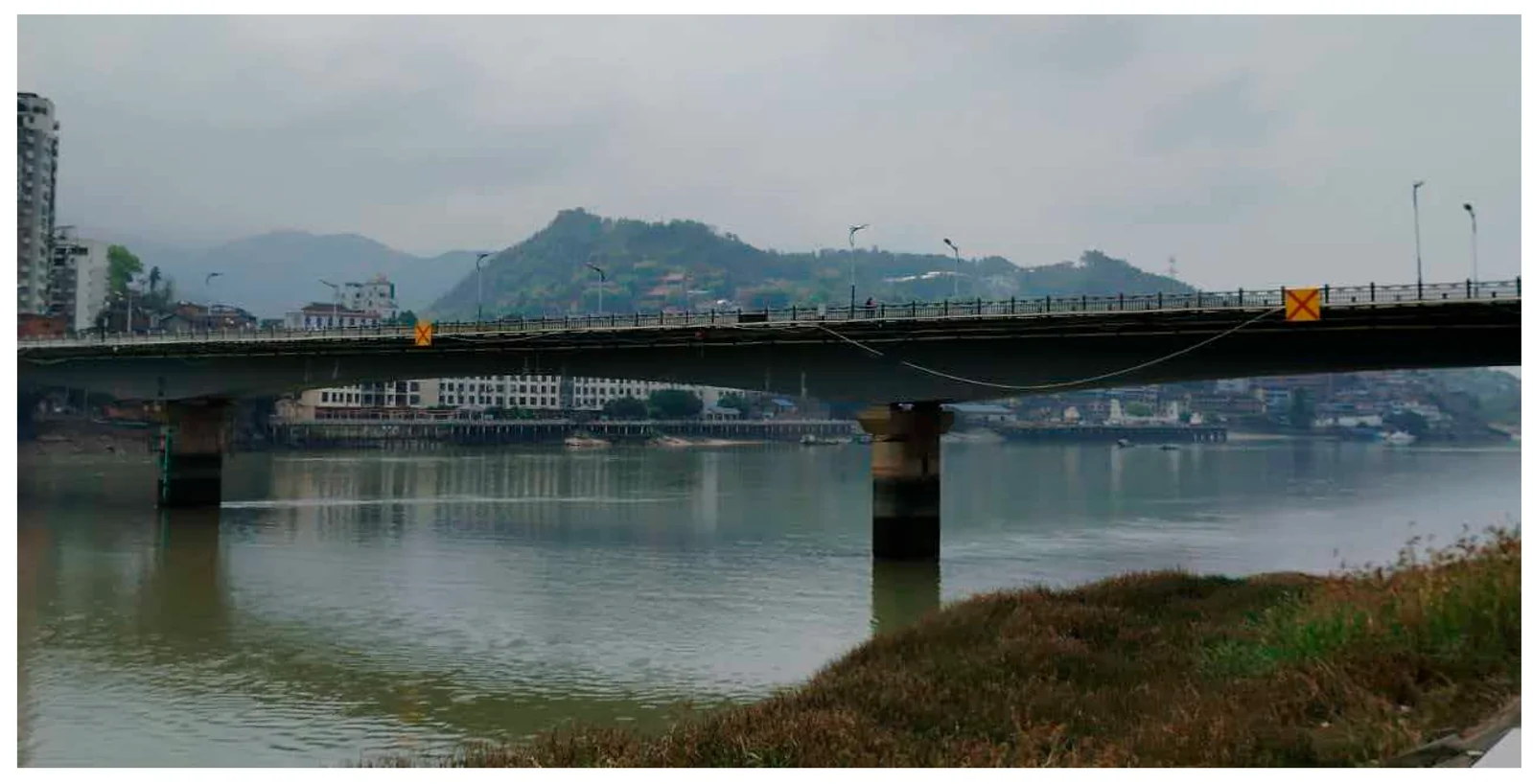
Photograph of Saiqi Bridge during the dry season.

**Figure 13:**
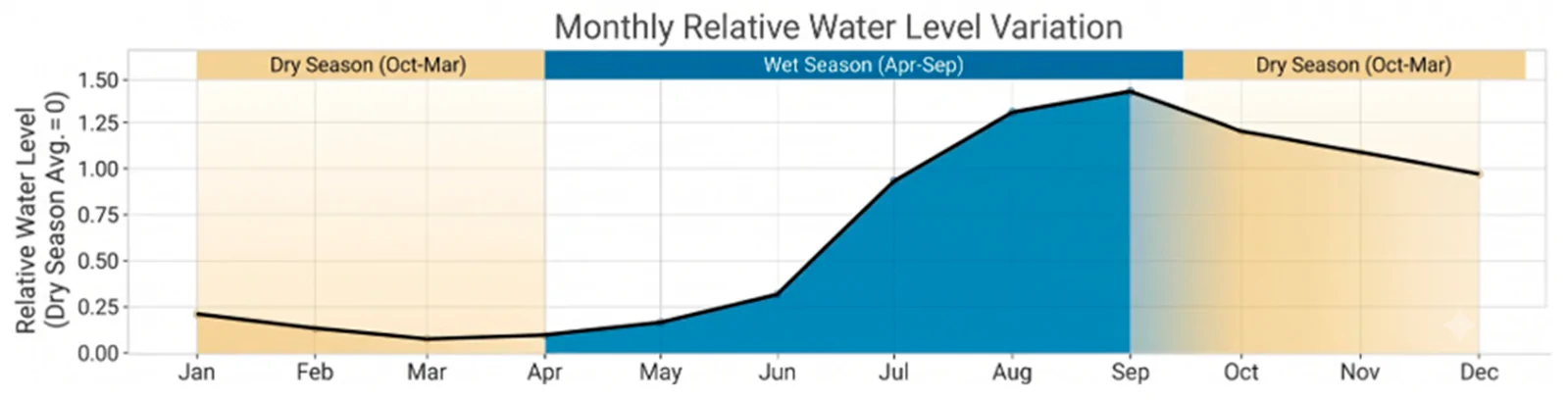
Statistical distribution of relative water levels at Saiqi Bridge.

To evaluate the practical applicability and structural relevance of the established constitutive model, a field-scale case study was conducted focusing on the concrete piers of the Saiqi Bridge. A general overview of the bridge infrastructure is provided in Figure 12. The hydrological environment at the bridge site is characterized by significant seasonal water level fluctuations, exhibiting a vertical differential of approximately 1.7 m between the dry and flood seasons. The statistical distribution of these historical water levels—which fundamentally govern the localized wetting–drying exposure regime experienced by the bridge piers—is graphically presented in Figure 13.

It should be noted that the natural exposure environment of the Saiqi Bridge differs from the controlled 10% Na_2_SO_4_ wetting–drying environment adopted in the laboratory. Therefore, the field investigation is used as an independent engineering-scale assessment of the proposed constitutive framework under long-term natural water-level fluctuations, rather than as a direct temporal counterpart of the 450-day laboratory exposure. The laboratory and field investigations thus provide complementary validation at the material and engineering scales, respectively.

The bridge piers are constructed using C40 concrete, where C40 corresponds to a characteristic cube compressive strength of 40 MPa according to the Chinese concrete strength-grading convention. The initial elastic modulus adopted in the present analysis is 32,500 MPa, with transverse reinforcement provided by spiral stirrups. To evaluate the current material state of the primary structural concrete, core samples were extracted from the main load-bearing regions of the pier shafts rather than from ancillary or protective concrete components. The sampling locations were selected to represent the structural concrete directly participating in the load-transfer path of the piers. These field samples exhibited an average in situ porosity of approximately 17%. Furthermore, after 25 years of continuous operational service, the actual peak compressive stress of the aged concrete was measured at 38.2 MPa.

No direct laboratory-to-field time equivalence or acceleration factor is assumed in the present study. The laboratory tests provide controlled validation under sulfate wetting–drying conditions, whereas the Saiqi Bridge serves as an independent field-scale validation of the model at an actual long-term degradation state.

Sampling Locations: To ensure the field validation is strictly relevant to the modeled environmental conditions, core extractions were exclusively targeted within the 1.7 m natural wetting–drying alternation zone. For each single-span pier, three representative cross-sections were selected: the lower bound (corresponding to the dry-season low water level), the mid-height of the fluctuation zone, and the upper bound (corresponding to the flood-season high water level). To ensure the acquisition of representative material properties, all core extraction sites were deliberately positioned to avoid congested reinforcement zones, honeycombed concrete, and any previously damaged or remediated regions. To ensure the acquisition of representative material properties, all core extraction sites were deliberately positioned to avoid congested reinforcement zones, honeycombed concrete, and any previously damaged or remediated regions. Within each designated cross-section, three spatially distributed measurement points were established. Core Sample Specifications: Intact concrete cores, measuring 50 mm in diameter and 50–100 mm in length, were extracted for laboratory analysis. To guarantee the reliability of the subsequent empirical testing, stringent quality control criteria were applied; only specimens completely devoid of surface cracks, edge chipping, and macroscopic internal voids were retained for evaluation. Sample Sealing and Preservation: Following extraction, all surface particulate matter and drilling debris were meticulously removed from the core samples. To preserve their in situ moisture content prior to testing, the specimens were immediately encapsulated in hermetically sealed containers. This preservation step effectively prevented both the evaporation of internal moisture and the absorption of exogenous water vapor from the ambient environment.

Test Surface Preparation: Prior to in situ testing, all target areas on the pier shafts were meticulously prepared. Surface contaminants—including dust, oil residues, degraded coatings, and loose mortar—were completely eradicated to yield a planar, dry, and structurally uniform testing surface.

Instrument Calibration: The moisture testing apparatus was initialized and calibrated in strict compliance with the manufacturer’s operational guidelines. To improve measurement accuracy and operational stability under field conditions, rigorous on-site zero-point calibration and ambient temperature compensation protocols were executed prior to data collection.

Point-by-Point Measurement: During data acquisition, the instrument probe was applied perpendicularly to the concrete surface, ensuring flush contact while strictly avoiding any interfacial gaps or eccentric loading. To ensure data reliability, each designated sampling location was subjected to three consecutive measurements, with only stable readings recorded for subsequent statistical evaluation.

Data Processing and Archiving: During the data reduction phase, any statistical outliers or anomalous readings were systematically excluded. The arithmetic mean of the three valid measurements was calculated to represent the definitive moisture content for each respective test point. Furthermore, all physical sampling coordinates were precisely marked, and the acquired datasets were systematically archived to support the comprehensive structural assessment.

**Figure 14:**
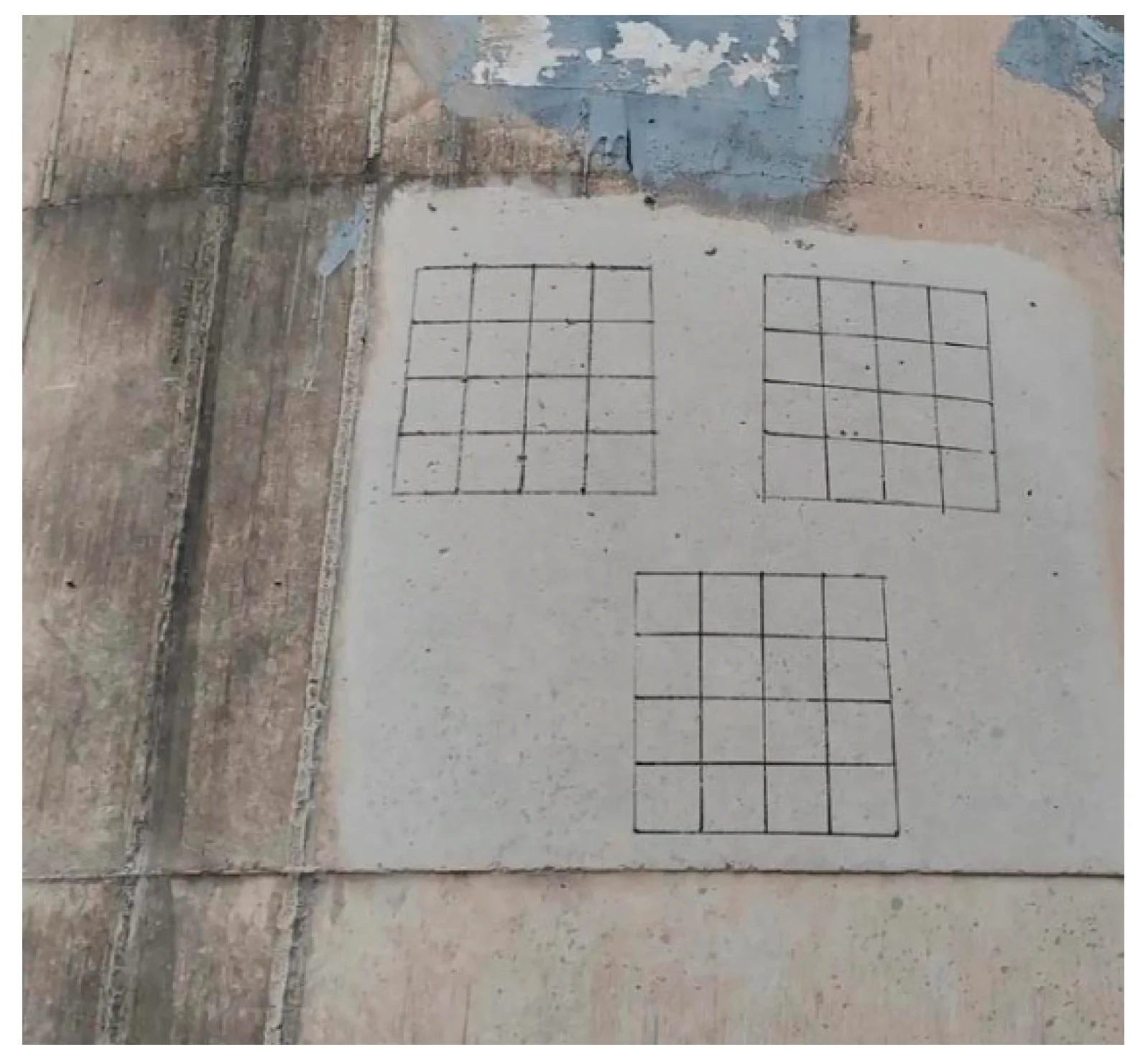
Layout of the non-destructive testing grid on the Saiqi Bridge pier cross-sections.

Test Grid Layout: A systematic testing grid was established by uniformly distributing sixteen 200 × 200 mm measurement zones across each designated pier cross-section (Figure 14). To preclude external interference and improve data reliability, the grid arrangement was meticulously positioned to avoid underlying reinforcement, embedded steel components, and localized surface defects such as macroscopic voids, cracks, or deteriorated regions.

Surface Preparation: All measurement grids were meticulously prepped by completely removing surface laitance, particulate dust, and oily contaminants. Localized topographical irregularities were lightly abraded with fine-grit sandpaper to achieve a highly planar finish, ensuring full and uniform contact between the rebound hammer plunger and the concrete surface.

Instrument Commissioning and Calibration: Prior to formal data acquisition, the rebound hammer was subjected to standard anvil verification and calibration. Only instruments demonstrating unimpeded plunger articulation, high reading repeatability, and an absolute absence of mechanical jamming or systematic bias were approved for field deployment.

Rebound Measurement: During testing, the instrument was maintained strictly orthogonal to the concrete surface and actuated with steady, uniform pressure. The corresponding rebound index was recorded immediately following each impact. A total of 16 consecutive impacts were executed within each designated test grid, with all empirical values comprehensively logged for analysis.

Carbonation Depth Measurement: Three spatially distributed test points were selected across each evaluated cross-section. A localized penetration was made through the concrete cover utilizing an impact drill, and all residual pulverized debris was meticulously extracted. A standard phenolphthalein indicator solution was subsequently applied to the freshly exposed internal substrate. The depth of carbonation was quantified as the orthogonal distance from the distinct color-change boundary to the exterior concrete face. The arithmetic mean of these three localized measurements was adopted as the representative carbonation depth for that specific cross-section.

Data Reduction and Correction: To mitigate the influence of localized anomalies and statistical outliers, the three highest and three lowest rebound indices from each grid were systematically discarded, retaining exactly 10 valid measurements for calculation. In strict accordance with prevailing non-destructive testing (NDT) specifications, the initial rebound-derived compressive strength was mathematically calibrated by integrating compensation factors for the measured carbonation depth, the instrument testing angle, and the specific condition of the concrete casting surface.

For in-service ordinary concrete bridge piers, a maximum surface moisture content of 5.0% is generally deemed acceptable under standard operational conditions. However, for structural elements slated for anticorrosion treatment or protective coating application, this threshold should be strictly maintained below 4.0% prior to the commencement of surface construction. Excessive residual moisture indicates inadequate drying of the concrete matrix, a condition known to precipitate subsequent durability defects such as surface dusting, micro-cracking, and premature delamination of protective coatings. Consequently, pier sections exhibiting non-compliant moisture levels must undergo extended ambient air drying, followed by mandatory re-evaluation, before any subsequent construction or remediation phases can be authorized.

**Figure 15:**
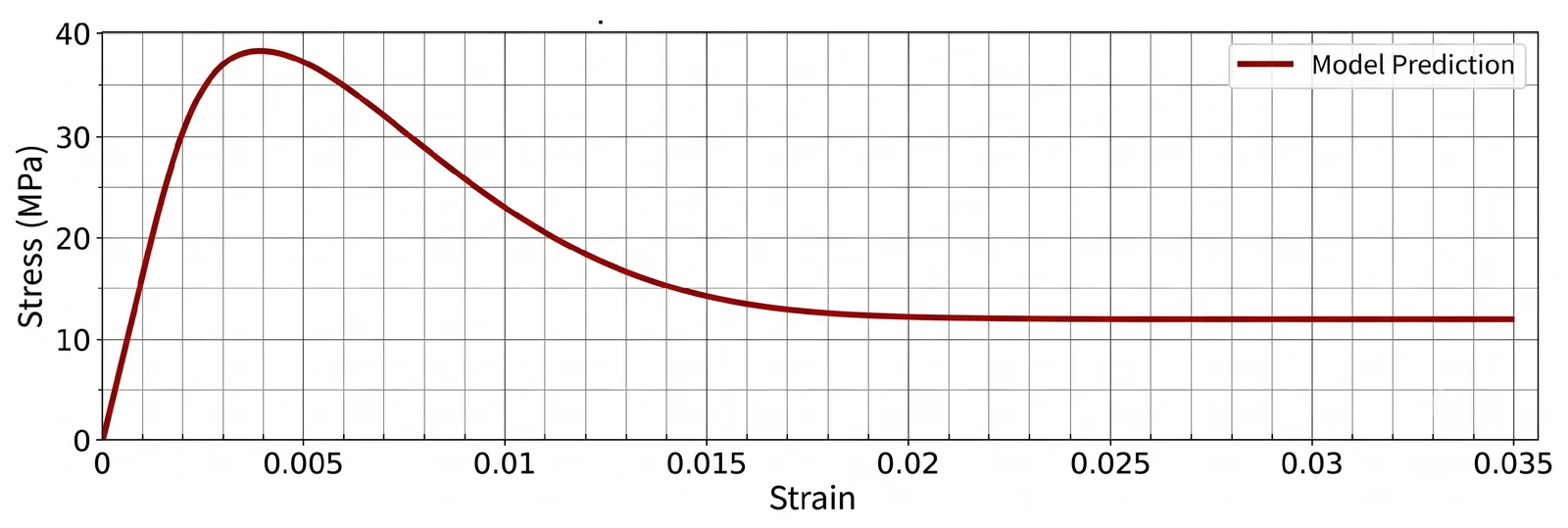
Analytically derived stress–strain response curve for the Saiqi Bridge pier concrete.

The evaluation criteria for in situ concrete compressive strength are established based on the following structural performance thresholds. Empirically measured strengths that meet or exceed the original design grade are classified as fully compliant. Conversely, measured values falling below the design threshold, but maintaining a deviation of less than 10%, are deemed marginally acceptable; however, structural components falling within this tier mandate continuous periodic monitoring. Strength deficiencies exceeding the 10% margin strictly fail to meet acceptance requirements. In such instances, a formal rectification and re-inspection protocol must be initiated to support a comprehensive structural safety reassessment. By incorporating the site-specific material parameters of the bridge into the proposed constitutive model, the resulting stress–strain response curve is analytically derived, as graphically depicted in Figure 15.

Analysis of the theoretical curve reveals that the peak compressive capacity of the pier concrete degrades under cyclic wetting–drying conditions, decreasing from the original design strength of 40 MPa to a residual strength of 38.2 MPa. This corresponds to a net structural capacity reduction of approximately 4.5%. Furthermore, the modeled stress-stress–strain accurately reflect the progressive increase in internal porosity induced by advancing cyclic environmental exposure. By performing an inverse analytical calculation using Equation (14) to back-calculate the porosity, a theoretical value of approximately 16.9% is derived. This theoretically predicted value exhibits a close agreement with the empirically measured in situ porosity of 17%. Ultimately, this high degree of correlation supports the applicability and practical applicability of the proposed porosity-dependent constitutive model for the structural assessment of in-service bridge infrastructure.

Although the back-calculated porosity of 16.9% exhibits excellent agreement with the measured average value of approximately 17%, this deterministic result must be interpreted within the context of field uncertainties. An intrinsic sensitivity exists within the inverse analytical calculation governed by Equation (14). For instance, if the field-measured residual peak strength (38.2 MPa) fluctuates by 5% due to spatial heterogeneity across the pier cross-sections or inherent tolerances in testing equipment, the theoretical undamaged matrix strength being constant, the back-calculated porosity would vary accordingly within a bounded range of approximately 12.7% to 21.1%. This sensitivity analysis highlights that while natural stochastic variations in in-situ conditions will introduce uncertainty into point-value predictions, the proposed model framework fundamentally remains robust, providing a reliable diagnostic envelope for structural condition assessment.

## 5. Discussion

The calibrated parameters  λ , β and *E*_0_ should be regarded as condition-dependent material parameters rather than universal constants. In the present study, they were determined for concrete subjected to sulfate wetting–drying exposure. Because these parameters are associated with the mechanical response of the material, changes in the degradation mechanism may lead to corresponding variations in their calibrated values. Therefore, although the general constitutive framework may be extended to other deterioration conditions, application to freeze–thaw cycling, chloride attack, carbonation, or coupled environmental actions requires recalibration and independent validation.

The present model should also be distinguished from a time-dependent environmental deterioration model. Environmental variables such as temperature, humidity, sulfate concentration, wetting–drying frequency, and exposure duration are not explicitly introduced as independent state variables. The model therefore describes the stress–strain response of concrete at a specified degradation state rather than directly predicting deterioration over a given service period. Future work should extend the validation to lower-porosity structural concrete and other degradation mechanisms, incorporate microstructural evidence of pore evolution, and further investigate environmental effects and post-peak behavior to improve the general applicability of the constitutive framework.

It is important to acknowledge that the current study relies predominantly on macroscopic phenomenological indicators to validate the proposed model. The evolution of the internal pore structure under prolonged sulfate wetting–drying cycles was indirectly inferred through macro-physical property changes, specifically the progressive variations in specimen mass and permeability coefficients. According to established multi-scale research, the observed initial mass gain corresponds closely to the deposition of expansive hydration products (e.g., ettringite and gypsum) within the existing voids, whereas the subsequent mass loss and permeability escalation strongly indicate crystallization-pressure-induced microcracking and enhanced pore connectivity. However, the lack of direct microstructural evidence—such as Scanning Electron Microscopy (SEM), X-ray Diffraction (XRD), and Mercury Intrusion Porosimetry (MIP)—limits a quantitative correlation between the microscopic damage mechanisms and the macroscopic statistical damage variable D. Therefore, future work should prioritize incorporating microstructural analysis techniques to track pore size distribution, morphology, and phase composition progressively. Integrating such multi-scale physical evidence will fundamentally strengthen the theoretical basis of the constitutive model. Furthermore, extending the validation to lower-porosity structural concrete and incorporating environmental variables into the constitutive formulation remain key objectives for advancing the framework’s general applicability.

## 6. Conclusions

Based on the strain equivalence principle and Weibull statistical framework, this study developed a porosity-dependent uniaxial compressive constitutive model and evaluated its applicability using controlled laboratory experiments and field data from the Saiqi Bridge. The main conclusions are as follows:

(1) Formulated by integrating the strain equivalence principle with Weibull statistical distribution theory, the proposed constitutive model describes the complex non-linear stress–strain behavior of concrete subjected to cyclic wetting-drying environments. Extensive validation against experimental data confirmed that the theoretically derived curves exhibit good agreement with the actual macroscopic mechanical responses, thereby substantiating the model’s predictive capability for evaluating concrete infrastructure subjected to axial compression within fluctuating water-level zones.
(2) The experimental results showed an initial mass increase followed by a gradual decrease, while the permeability coefficient generally increased with wetting–drying exposure and initial porosity. These observations provide complementary evidence of the progressive physical deterioration of the specimens.
(3) The practical engineering applicability of the proposed model was further evaluated through a full-scale structural assessment utilizing core samples extracted from the 25-year in-service Saiqi Bridge. The theoretical framework reproduced the long-term strength degradation of the pier concrete, modeling the reduction from the initial design strength of 40 MPa to a field-measured residual capacity of 38.2 MPa (representing a 4.5% structural decline). Furthermore, an inverse analytical calculation performed using the model derived a theoretical concrete porosity of 16.9%, which exhibits good agreement with the empirically measured in-situ porosity of 17%.
(4) By incorporating pore-related characteristics into the constitutive formulation, the proposed model provides a simplified analytical framework for evaluating the compressive response of concrete at a given degradation state. The model can support the mechanical assessment and numerical analysis of concrete structures subjected to wetting–drying exposure. Further validation under broader concrete types and environmental conditions is required to improve its general applicability.

## Figures and Tables

**Table 1:** Mix proportions of the pervious concrete mixtures.

No.	Target Porosity (%)	Cement (kg/m^3^)	Aggregate (kg/m^3^)	Water (kg/m^3^)	Superplasticizer (kg/m^3^)	SR Binder (kg/m^3^)	Steel Fiber (kg/m^3^)	Remarks
S1	15	485.3	1438	120	1.92	0	0	Ordinary type
S2	15	467.5	1438	117	1.86	23.76	0.86	Reinforced type
N3	20	428.2	1438	108	1.72	0	0	Ordinary type
N4	20	429.7	1438	107	1.71	19.25	1.7	Reinforced type
W5	25	368.3	1438	92	0.85	0	0	Ordinary type
W6	25	368.1	1438	92	1.11	15.33	2.6	Reinforced type

**Table 2:** Permeability coefficient test results of pervious concrete specimens.

No.	Actual Porosity (%)	Average Permeability Coefficient (cm/s)	Remarks
S1	14.88	0.31	Ordinary type
S2	15.02	0.32	Reinforced type
N3	19.75	0.45	Ordinary type
N4	20.15	0.47	Reinforced type
W5	24.67	0.53	Ordinary type
W6	25.21	0.54	Reinforced type

**Table 3:** Calibrated model parameters and ascending branch constitutive equations for each specimen group.

No.	Actual Porosity (%)	Average Permeability Coefficient (cm/s)	*E*_0_	*λ*	Ascending Branch Constitutive Equation
S1	14.88	0.31	2780	1.847	σ = 3264.83 εexp(−0.541(ε/2.01)^1.847^)
S2	15.02	0.32	2780	1.568	σ = 3272.90 εexp(−0.638(ε/2.34)^1.568^)
N3	19.75	0.45	2780	1.769	σ = 3464.61 εexp(−0.565(ε/1.82)^1.769^)
N4	20.15	0.47	2780	1.458	σ = 3480.66 εexp(−0.686(ε/2.04)^1.458^)
W5	24.67	0.53	2780	1.652	σ = 3666.10 εexp(−0.637(ε/1.57^1.652^)
W6	25.21	0.54	2780	1.385	σ = 3725.54 εexp(−0.568(ε/1.76)^1.385^)

**Table 4:** RMSE and R^2^ values between the model-predicted and experimental stress–strain curves for the six specimen groups.

No.	RMSE	R^2^
S1	0.456	0.9966
S2	0.107	0.9998
N3	0.101	0.9998
N4	0.300	0.9986
W5	0.267	0.9989
W6	0.413	0.9973

## Data Availability

The original contributions presented in this study are included in the article. Further inquiries can be directed to the corresponding authors.
